# A Targeted Survey for Scrapie in Jos Plateau State, Nigeria

**DOI:** 10.1155/2013/841978

**Published:** 2013-07-31

**Authors:** O. O. Nwankiti, E. I. Ikeh, O. A. Arowolo, A. J. Nwankiti, M. O. Odugbo, T. Seuberlich

**Affiliations:** ^1^National Veterinary Research Institute, PMB 1 Vom, Plateau State 930010, Nigeria; ^2^Department of Medical Microbiology, School of Medicine, University of Jos, PMB 2084, Jos 930001, Nigeria; ^3^NeuroCentre, National and OIE Reference Laboratories for BSE and Scrapie, Division of Neurological Sciences, Vetsuisse Faculty, University of Berne, 3001 Berne, Switzerland

## Abstract

Scrapie, a disease of sheep and goats with a progressive course and fatal outcome, has not been identified in Nigeria. Anecdotal scrapie reports by livestock workers abound. Livestock diseases like scrapie form huddles in livestock economics of countries. For 8 months we surveyed for scrapie targeting emergency/casualty slaughter sheep and goats in Jos, Nigeria. We clinically examined 510 sheep and 608 goats of local breeds, aged from 12 months to 5 years. In total 31 (5.10%) goats and no sheep were clinically suspicious for scrapie. Caudal brainstem tissues of suspect animals collected *postmortem* were analyzed for the disease specific form of the prion protein, PrP^Sc^, using Bio-Rad's TeSeE ELISA rapid test kit. No sample was positive for scrapie. Fluorescent antibody test for rabies and H&E staining on samples were carried out for differential diagnosis. These showed no pathological lesions indicative for neurological disease. While our findings do not exclude the presence of scrapie in Jos, we demonstrate that targeted sampling of small ruminants for neuroinfectious disease is feasible in developing countries, pointing to the possibility of implementing such a monitoring scheme in Nigeria to prevent economic losses in small ruminant livestock as scrapie caveats from endemic countries have shown.

## 1. Introduction

Scrapie is a naturally occurring progressive, fatal, infectious, and neurodegenerative disease of sheep and goats that has been recognized for over 250 years. The occurrence of scrapie preceded the recognition of other prion diseases of mammals and so can be referred to as the archetype of prion disorders [1]. It is caused by an infectious misfolded protein, termed prion. Like other TSEs, this misfolding is thought to be caused by conformational change of the normal host prion protein (PrP^C^) mediated by the presence of the abnormal, misfolded prion protein (PrP^Sc^). Host PrP variations are known to play a role in the susceptibility of goats and sheep to scrapie [2–4]. It has been proposed as the root from which bovine spongiform encephalopathy (BSE) emanated [5]. Scrapie is now considered a worldwide disease of small ruminants and currently represents a significant part of the detected transmissible spongiform encephalopathies (TSEs). 

The estimated population of sheep and goats in Nigeria is 27 million, and 40.8 million respectively [6]. Sheep are mainly kept for subsistence and religious inclinations while certain goat species are kept for food and high quality leather production [7]. Livestock diseases pose a challenge to this growing economic sector. Scrapie has not been identified so far in Nigeria and research on scrapie and indeed other prion diseases in Nigeria is virgin. However, anecdotal reports of clinical suspicious scrapie and other neurologic trans-boundary diseases abound. Our study aims to establish a basis for an active surveillance programme that primarily addresses the question on the presence of scrapie in Nigeria. This is intended to give an overview of what a wider survey may reveal.

## 2. Area Descriptions, Material Studied, Methods, and Techniques

This study was carried out in Jos, Nigeria, which is a hub of livestock traversing the middle belt and northern parts of the country. We initially planned to sample cases of fallen stock and scrapie suspects from the field where unconfirmed reports of scrapie filter in once in a while. This proved difficult due to the uncooperative attitude of livestock owners/herds men and poor records on their animals. We noted that most diseased animals that would match the clinical picture of scrapie ended up in the abattoir for the food chain. Hence, we targeted samples of emergency or casualty slaughtered sheep and goats. We examined sheep and goats brought in for emergency or casualty slaughter at the abattoir for clinical signs of scrapie [8] using the guidelines in the OIE Terrestrial Animal Health Code 2009 [1]. For this study, we defined emergency/casualty slaughter animals as sheep or goats that are brought in for slaughter due to unidentified disease condition or debilitating condition that the owner or veterinarian could not identify or treat successfully. All animals brought in as routine slaughter are not inclusive.

Sampling was from January to August 2011. 510 sheep and 618 goats aged from 12 months to 5 years were clinically examined. Physical appearance and dentition were used to assess the animals age. The clinical examination included the assessment of changes in mental status, behavior and activity, pruritic activities, postural changes, gait changes, involuntary movements, weight loss, and loss of condition. Our criteria for selecting clinical scrapie suspect animals primarily focused on animals that showed pruritis, increased levels of excitement on sudden agitation coupled with ataxia or discordant movement, and signs in other categories we could examine for (see Tables 1 and 2; criteria used for case-definition are indicated by italic figures).

We collected caudal brain stems from clinical suspect animals through the foramen magnum *postmortem*, using a special harvesting spoon. From each sample, a 1 g portion and two 2 g portions of the obex region were cut out. The first 2 g portion was fixed in a sample bottle in 10 times its volume of formalin. Parts of the fixed portions were used for histological processing and hematoxylin and eosin (H&E) staining while the remaining part was left for confirmation of rapid test positive samples via immunohistochemistry (IHC) [1]. The second portion was packed in a transport container and stored frozen −20°C, likewise the remaining portion of the brain stem. The 2 g frozen portions were shipped to the NeuroCentre, University of Berne, Switzerland, for scrapie testing. The 1 g portions were used for rabies diagnosis via indirect fluorescent antibody testing (DFA) technique. 

Testing for scrapie was done using the TeSeE ELISA purification kit (Bio-Rad, ref.: 355 1165) and detection kit (Bio-Rad, ref.: 355 1166). This kit employs an immunoenzymatic technique (sandwich format) using two monoclonal antibodies for the detection of PrP^Sc^. While the type of these monoclonal antibodies is subject to industrial secret, its capacity to detect classical scrapie in a range of sheep with different PrP genotypes has been shown [9]. However, similar data for goats are still missing. This test was carried out according to the manufacturers guide after sample preparation/clarification and proteinase K digestion. Any positive sample would have been confirmed by IHC PrP^Sc^ staining method using monoclonal antibody F99 (VMRD, epitope at the prion protein C-terminal: QYQRES) on tissue sections prepared from formalin fixed tissues [1]. Scrapie positive and negative controls were set up alongside kit controls during testing. We did not determine the PrP genotype of the clinical suspicious goat samples collected before screening for scrapie because of limitations in funding.

## 3. Results and Discussion

By clinical examination, we did not identify any scrapie suspect sheep (Table 1) but identified in total 31 (5.10%) of 608 goats as scrapie suspicious (Table 2). The absence of clinically suspicious sheep may be attributed to the religious importance attached to sheep by the predominant tribe (Fulani) that breed sheep as compared to goats in the study area. Apparently healthy sheep with mild/visible debilitation that could be overlooked were brought in for emergency slaughter unlike goats that were basically sold and slaughtered for food and leather. Hence on examination and in the absence of pruritis and gait problems we classed such sheep as clinically unsuspicious though they were examined for some other signs (Table 1). We note that nonoptimal or ideal *ante mortem* livestock handling practices by owners/butchers prior to slaughter may have elicited increased agitation. These could have led to poor clinical examination of some sheep and goats which may have given rise to appearances of seemingly clinical suspects.

Clinical examination revealed 31 goats with neurological signs indicative for scrapie. However, this suspect could not be confirmed by laboratory testing. This underlines the limitations associated with clinical examination of livestock for prion disease [10], as clinical signs in scrapie are not very specific. 

The PrP genotypes of local breeds of goats and sheep in Nigeria are yet to be determined. As the nature and proportion of PrP genotypes in the sheep and goat population in Nigeria remain unknown, it is possible that previously unnoticed PrP genotypes are present that interfere with the performance of diagnostic tests applied but this remains unknown. Additional studies are required to fill this gap of knowledge. For differential diagnosis, it may be worthwhile to mention that we did not specifically examine nor sample target animals for other diseases like listeriosis, tetanus, brain worm (Cerebrospinal Nematodiasis), caprine arthritis, and encephalities [11] that may have given differential diagnosis viz a vis neurological diseases of small ruminants. We only carried out rabies diagnosis and H&E staining because these tests can be done using the same samples collected for scrapie testing. None of the samples tested positive for rabies, ruling out rabies as an underlying neurological disease. The H&E pictures did not reveal any TSE pathological conditions which appear as characteristic vacuolation affecting the neuropil with an anatomical and bilateral symmetrical distribution [12]. This means that the observed seeming neurological conditions that we aligned with discordant movement or other abnormal gait conditions may be results of other, not neurological disease. Pruritis may have been results of early infection with dermatophilic diseases that were not visible and had not degenerated to loss or discolouration of fur. Considering that we used a targeted sampling method which excluded other surveillance streams, a small sample size and area of study in relation to the sheep and goat population of the country, our findings should not be seen as the absence of scrapie in the study area or extrapolated as an extended picture of the countries scrapie status. We have only shown that sheep and goat that we considered clinical suspect cases alongside anecdotal reports were not positive for scrapie using TeSeE ELISA by Bio-Rad testing and the criteria we used for clinical suspicion may have been misinterpreted or not properly applied.

Difficulties and challenges that may be faced when establishing an active surveillance in Nigeria are some we came across in this study: the nomadic nature of livestock farming, poor enlightenment, poor or inexistent records of livestock by owners, no compensatory benefit systems in place for livestock owners and inadequate veterinary services. These affect the quality of demographic data obtained and ease of diagnosis. Clinical suspect animals are not being officially reported for testing, hence an alternative means is to actively search for animals with neurological signs as we did until the challenges confronting the proper setup of a surveillance system for TSE are addressed; enlightenment programs for farmers and animal handlers, personnel training on prion diseases and proper laboratory diagnostic setups in place by government agencies concerned. 

This study lays the groundwork for an efficient and cost-effective surveillance of scrapie and other neuroinfectious diseases of livestock in resource-limited countries, such as Nigeria. This we have demonstrated by systematic *ante mortem* inspection of emergency and casualty slaughter animals that would have identified neurologically diseased small ruminant livestock in a relatively high proportion. We advocate the use of our findings as a platform for the setup of an active surveillance for scrapie and other animal TSEs in Nigeria so as to determine the scrapie status for economic reasons. 

## Figures and Tables

**Table 1:** Results of clinical examination and laboratory testing of emergency/casualty slaughter sheep for scrapiefrom Jos, Nigeria.

	Number of sheep per breed and sex								Total
	Yankasa		Uda		West African dwarf		Balami		
	Male	Female	Male	Female	Male	Female	Male	Female	
Clinical examination	250	20	117	13	59	19	17	15	510

Changes in mental status, behaviour and activity									
Self-Isolation									0
Disobeys herdsman's calls									0
Flighty on approach									0
Hyperalert	5								5
Increased response to external stimuli									0
Somnolent/dull/vacant gaze/lowered head	50	15	11	8	45	11	8	10	150
Often lies down	16	3	10	2	7	18	11	2	69
Reduced social interaction	50	15	11	8	45	11	8	10	150
Frequent teeth grinding									0
Loss of appetite	33	17	11	6	40	11	5	7	130
Pruritic activities									
Increased grooming behaviour									0
Rubs on objects frequently									0
Licking/smacking of mouth									0
Nibble reflex response									0
Rapid tongue extrusion									0
Poor response to scratch test									
Body discolouration									0
Postural changes									
Lowered hind with flexed limbs									0
Gait changes									
Ataxia/discordant movement									0
Involuntary movements									
Head tremors									0
Seizures									nil
Weight loss									
Weight loss	57	18	51	12	45	10	15	13	221

Clinically suspicious									0

**Table 2:** Results of clinical examination and laboratory testing of emergency/casualty slaughter goat for scrapie, rabies, and H&E from Jos, Nigeria.

	Number of goats per breed and sex								Total
	Kano brown		Sokoto red		West African dwarf		Sahel/Desert		
	Male	Female	Male	Female	Male	Female	Male	Female	
Clinical examination	200	94	182	5	77	16	20	14	608

Changes in behaviour and activity									
Self-Isolation	33	12	7			3			55
Disobeys herdsman's calls									0
Flighty on approach	*37 *	15	*12 *	5	*17 *	*6 *	*3 *	*5 *	100
Hyperalert	*10 *	6	*5 *	*4 *	*4 *	*3 *			32
Increased response to external stimuli	*10 *	6	*5 *	*4 *	*4 *	*3 *			32
Somnolent/dull/vacant gaze/lowered head									0
Often lies down	3	1		2		1			7
Reduced social interaction	3	1		2		2			7
Frequent teeth grinding									0
Loss of appetite	45	13	27	5	21	8	5	3	127
Pruritic activities									
Increased grooming behaviour	*11 *	*6 *	*5 *	*3 *		*5 *			29
Rubs on objects frequently	*8 *	*21 *	*7 *	*1 *	*5 *	*7 *	*1 *	*1 *	51
Licking/smacking of mouth	14	13	5			*2 *			34
Nibble reflex response									0
Rapid tongue extrusion	14	13	5			*2 *			34
Poor response to scratch test									0
Body discolouration									0
Postural changes									
Lowered hind with flexed limbs	*10 *		*6 *	*2 *	*10 *	*2 *	2	2	35
Gait changes									
Ataxia/discordant movement	*8 *		*7 *	*2 *	*9 *	*2 *	2	1	31
Involuntary movements									
Head tremors	*7 *		*5 *			*1 *	1	1	15
Seizures									
Weight loss									
Weight loss	53	47	71	5	23	15	11	7	238

Clinically suspicious	8		7	2	9	2	2	1	31

TeSeE ELISA test positive									0

DFA test positive									0

H&E test result									0

Italic figures denote parameters considered for selecting suspects.
